# Contextualising migrants’ health behaviour - a qualitative study of transnational ties and their implications for participation in mammography screening

**DOI:** 10.1186/1471-2458-13-431

**Published:** 2013-05-03

**Authors:** Linnea Lue Kessing, Marie Norredam, Ann-Britt Kvernrod, Anna Mygind, Maria Kristiansen

**Affiliations:** 1 Danish Research Centre for Migration, Ethnicity, and Health (MESU), Section for Health Services Research, Department of Public Health, University of Copenhagen, Copenhagen, Denmark; 2 Department for Cancer Prevention and Documentation, The Danish Cancer Society, Copenhagen, Denmark; 3 Section for Social Pharmacy, Department of Pharmacy, Faculty of Health and Medical Sciences, University of Copenhagen, Copenhagen, Denmark

**Keywords:** Migrant women, Mammography screening, Contextualised, Health and social issues, Transnational ties

## Abstract

**Background:**

Lower participation rates in mammography screening are common among migrant women compared to native-born women. Explanations of these lower rates have mainly been based on behavioural theories investigating how lack of knowledge, access to services and culture influence the screening behaviour. The aim of the present study was to contextualise screening behaviour by exploring migrants’ transnational ties and their influence on participation in mammography screening in Denmark.

**Methods:**

The study is based on the analysis of qualitative interviews with 29 women residing in greater Copenhagen, Denmark and born in Somalia, Turkey, India, Iran, Pakistan and Arab-speaking countries.

**Results:**

We found that while women had knowledge about breast cancer and mammography screening, it was not prioritised. All women were embedded in transnational ties, which they struggled to retain through emotional and financial obligations, and these current struggles in their everyday life seemed to leave little room for concerns about breast cancer and therefore seemed to contribute to their lower participation in screening.

**Conclusions:**

The study emphasises the need to take into account the multi-layered and multi-sided factors in migrants’ everyday life in order to further understand their health behaviour.

## Background

Numerous studies have documented lower participation in mammography screening among various migrant groups [1-3]. A recent Danish study shows that while 74% of Danish born women participate in mammography screening 61% of non-Western migrant women attend screening [3]. Determinants of non-use among migrants, which have been identified, relate to psychosocial, demographic and socioeconomic factors. These determinants, which reduce participation in mammography screening, include, lack of knowledge, trust, language barriers, low socio-economic status, concerns regarding mammography screening, embarrassment and pain [2,4-6]. Moreover, the influence of social support, for example help with translating the invitation, transportation to “screening site” and emotional support when waiting for the results, has been found to influence participation [3,6].

Recent studies have criticised previous research for focusing mainly on direct influences on behaviour such as the psychological characteristics of individual migrant women [7-11]. For example, a review of theories regarding mammography screening shows that behavioural theories have been uncritically applied across race and ethnic groups without taking into account the context within which these groups are situated within [12]. Contextual aspects can be categorised based on: 1) *the immediate social context* (family interactions, neighbourhood, and community relationships and support); 2) *the contextual realm of institutions and organisations* (the structuring of access and barriers to health care and the ability to mobilise resources to get what one needs); and 3) *the political, historical and legal realm* (historical memories, experiences of migration, discrimination, colonisation, and decolonisation, as well as large-scale demographic, politico-economic, cultural, and historical factors, laws, regulations, and policies) [7]. There is a need to move beyond individual psychological characteristics and contextualise health behaviour in order to provide a more comprehensive understanding of how multiple aspects within migrants’ everyday lives may directly or indirectly affect their participation in mammography screening [7].

The relationship between migrants’ social relations and access to health care in the receiving country has recently been given more attention in studies on migrants’ participation in mammography screening [3,6,13-15]. Much less attention has been given to the influence of migrants’ transnational ties on mammography screening [9,16]. The present study recognises both the influence of psychological characteristics of the individual as well as migrants’ social relations on access to health care in the receiving country. Moreover, the present study argues that to further our understanding of migrants’ health behaviour these influences should be situated in a broader contextual setting, which includes the likely influence of transnational ties.

Migrants’ transnational ties make their social context different from that of other vulnerable groups and the general population. While the process of moving by definition means changes in geographical location, most migrants retain ties to their social relations in the country of birth and to relatives settling in other countries [17,18]. Thus, migration cannot be perceived as a one-way movement resulting in the gradual integration of migrants into the receiving country [19]. Rather it is imperative to study both the structure of transnational ties, for example the size (number of network members), density (the extent to which members are connected to each other) and the resources (emotional and financial obligations) [19]. Differences in individual factors may, however, affect migrants’ possibilities of forming and maintaining ties. Such factors could be the type of migration as well as legal structures in the receiving countries, regarding settlement regulations influencing the possibility of family reunification. Furthermore, the struggle to maintain these ties may influence migrants’ everyday lives and have an impact on their health behaviour.

The aim of the present study was to explore migrants’ transnational ties and their influence on participation in mammography screening in Denmark. We argue that transnational ties are essential to further understand migrants’ health behaviour, in the present study exemplified by screening behaviour. Transnational ties should be understood as efforts to maintain connections between *multiple* geographic locations (receiving country, country of birth, and other countries where relatives have settled).

## Methods

In this qualitative study, semi-structured interviews were performed with women whose country of birth was Somalia, Turkey, India, Iran, Pakistan and Arab-speaking countries. All participants resided in greater Copenhagen, Denmark at the time of the study. The women’s countries of birth represent some of the major well-established migrant groups in Denmark who come mainly as refugees or through family reunification.

Participants differed in regards to previous participation in the free organised mammography screening programme, which takes place in Copenhagen. The programme is offered biennially to women between 50–69 years and takes place at public hospitals. The programme is by letter invitation and not through general practitioner referrals. Women who are often categorised as socially marginalised were recruited due to the focus on the importance of social context. Socially marginalised women were defined as women living in socially disadvantaged neighbourhoods, multiethnic communities characterised by large unemployment rates and high crime rates.

From September to November 2011, we interviewed 29 women between the ages of 50–69 years (three women were slightly younger and two women were older) who had lived in Denmark from three to 40 years. All of them suffered from one or more diseases such as hypertension, diabetes, heart problems, arthritis, or depression (Table 1). The majority of participants had only a primary school education. After obtaining informed consent, the first and last author of this article carried out the interviews, eight individual interviews and six focus group interviews. Focus groups and individual interviews were combined in order to gain insight into how values, opinions and identities are constructed, developed and negotiated within social relations (focus group) as well as examining in-depth life stories through individual interviews [20,21].

**Table 1:** Demographic information

**Name**	**Age**	**Country of birth**	**Years in Denmark**	Residence permit*	Household**	Use of mammography screening***	**Co-morbidity**	**Family history of cancer**
Aida	59	Palestine	28	Refugee	Yes	Irregular user	Missing data	Yes: Lung
Zainab	55	Pakistan	32	F.R.	Yes	Non-participant	Diabetes	No
Meenu	60	India	35	F.R.	Yes	Non-participant	Hypercholesterolemia	No
Sumaiya	64	Somalia	21	Refugee	Yes	Non-participant	Heart failure, asthma, liver infection	Yes: Missing data
Layla	66	Somalia	Missing data	Refugee	No	Irregular user	Diabetes, arthritis, hypercholesterolemia	Yes: Missing data
Azmina	50	Turkey	32	F.R.	Yes	Opportunistic screening	Migraine	Yes: Breast
Reyhan	50	Turkey	12	F.R.	No	Opportunistic screening	Depression	Yes: Prostate, throat, breast
Sila	56	Turkey	15	F.R.	No	Opportunistic screening	Brain tumour, epilepsy	Yes: Brain, breast
Cherifa	55	Morocco	39	F.R.	No	User	Missing data	No
Ahlam	64	Morocco	3	F.R.	Yes	Missing data	Missing data	Yes: Brain, throat
Naziha	66	Morocco	40	F.R.	No	Irregular user	Missing data	Yes: Breast
Salma	49	Morocco	10	F.R.	Yes	Outside target group	Missing data	Yes: Breast
Fatima	57	Jordan	Missing data	F.R.	Missing data	Missing data	Missing data	Missing data
Afra	78	Palestine	Missing data	Refugee	No	Outside target group	Missing data	Yes: Liver
Abir	55	Iraq	11	F.R.	Yes	Opportunistic screening	Hypothyroidism, slipped disc, hypercholesterolemia	No
Malak	52	Jordan	19	F.R.	Yes	Opportunistic screening	Breast cancer, osteoporosis, arthritis, hypercholesterolemia	Yes: Ear
Yasmin	90	Morocco	9	F.R.	Yes	Outside target group	Diabetes, arthritis, hypercholesterolemia, Parkinson’s disease	Yes: Kidney
Aisha	50	Morocco	29	F.R.	Yes	No letter	Migraine	No
Meenu	60	India	35	F.R.	Yes	Non-participant	Hypercholesterolemia	No
Nadia	Missing data	Pakistan	37	F.R.	No	Non-participant	Diabetes, arthritis	No
Saima	47	Pakistan	20	F.R.	Missing data	Outside target group	Missing data	Yes: Missing data
Afareen	52	Iran	22	Refugee	No	Opportunistic screening	Missing data	No
Haleema	53	Pakistan	37	F.R.	Yes	User	Diabetes, allergy, arthritis, Hypothyroidism	Yes
Waris	58	Somalia	15	F.R.	Yes	Opportunistic screening	Diabetes	No
Nadifa	48	Somalia	11	F.R.	Yes	Outside target group	Missing data	No
Ifra	60	Somalia	16	F.R.	Yes	Irregular user	Migraine	No
Sofia	60	Somalia	12	F.R.	Missing data	Irregular user	Missing data	No
Isra	65	Turkey	21	F.R.	Yes	Irregular user	Allergy	Yes: Liver
Nur	67	Turkey	25	F.R.	Yes	Irregular user	diabetes, hypertension	Yes: Liver, lung

*F.R = Family Reunified.

** Yes = Living together with others. No = Living alone.

*** Irregular user: Had participated in the mammography screening programme once or more than once but not regularly every second year. User: Had participated in the mammography screening programme regularly every second year. Non-participant: Had never participated in the mammography screening programme. Opportunistic screening: Had undertaken a mammography screening outside of the programme. Outside target group: Had not received the invitation for mammography screening yet. No letter: Had not received the invitation for mammography screening yet even though being within target group.

Participants were recruited through contact persons in residential areas with high numbers of migrants, a mosque, and drop-in centres for pensioners and migrants. Additionally, snowball sampling was used. The women were introduced to the study both orally and by a written invitation in Danish, which explained the purpose of the study and issues concerning ethics and rights, such as anonymity and the right to withdraw from the study at any time. Before the interview participants were informed that interviews would be audiotaped and transcribed and that all identifying information would be deleted from the transcripts. This study received ethical approval by the Danish Data Protection Agency, the national agency securing storage and use of information about individuals.

The interview guide focused on knowledge, attitudes and practices regarding the healthcare system in general and mammography screening in particular. In order to contextualise mammography screening behaviour, questions regarding daily life, social networks, socioeconomic status and ties to country of birth were included. The interview guide was adjusted throughout the study.

The interviews took place in a location chosen by the women, either in public places or in private homes and lasted for 30–90 minutes. Notes about the interview and other impressions were taken immediately after the interview and used in the analysis as contextual information. Professional interpreters were used when needed. Interviews were transcribed verbatim in Danish and validated by a second interpreter. The citations presented here were translated from Danish to English. At the end of each interview, a short questionnaire was handed out containing questions on demographics, socioeconomic position and health status. Both interviewees and contact persons were acknowledged with a gift basket.

### Data analysis

The data were analysed through multiple readings of interview transcripts focusing on the depth of the material. Analytically, the focus was on expressions of well-being, everyday life in Denmark and ties to country of birth. During this process of analysing the material, themes related to possible barriers for participation in mammography screening were identified. The analytical approach was phenomenological, which emphasises gaining insight into the lived meanings of a phenomenon by meeting it as unprejudiced as possible [22,23]. The first and last author carried out the analyses independently and later compared their findings. Firstly, all transcripts were read through in order to gain an impression of the text as a whole. Secondly, meaning units were identified across interviews and major themes abstracted. Thirdly, the essence of the particular theme was synthesised into a consistent statement across interviews, thereby moving from concreteness to a more abstract level of understanding. Findings were discussed with researchers of cross-disciplinary backgrounds.

Two main analytical concepts were used to contextualise migrants’ health behaviour. We used the concept of *social context* to explore how multiple interwoven aspects in the women’s everyday lives may influence their participation in mammography screening directly and indirectly [7]. *Transnational ties* was used to explore more thoroughly the women’s social and economic ties as both multi-layered and multi-sited [18,19]. The analytical lens was on the structure and resources of the women’s transnational ties and their implications for the women’s health behaviour in relation to mammography screening.

## Results

The results are presented in two parts. First a brief description of the women’s perceptions of and attitudes towards mammography screening mostly in correspondence with the well-know barriers for participation in mammography screening. The second part is an illustration of how the women’s embeddedness in transnational ties further influenced their participation in mammography screening directly and indirectly.

### Perceptions of and attitudes towards mammography screening

The women had some knowledge about breast cancer and perceived the disease as dangerous. Many had a family history of cancer; five had specific experiences with breast cancer within the family and mentioned risk factors such as stress, genetics and health behaviour as causes of the disease. Participants discussed multiple health problems such as hypertension, diabetes, heart problems, and arthritis and articulated that they were in regular contact with their general practitioner. In general, women showed a high degree of trust in and satisfaction with the Danish healthcare system.

All women were aware of the existence of the mammography screening programme. More than half of them had a mammogram at one time, but only two participated regularly in the programme. Some women who did not participate in the programme articulated a wish to participate. Naziha from Morocco said:

“You see it is because it is a good thing to attend examinations and see how it looks (…)”. (Naziha)

Well-known barriers were mentioned by some of the women such as inability to read the invitation, lack of transport to “screening site” and lack of emotional support when waiting for the results. Aida, born in Palestine, had a screening when she was younger and explained the importance of social support:

“I started to have pain here [pointing at her chest], and I was afraid that it was cancer. And my son pushes me, he said: “you have to go” and we went together to see it [have it examined]”. (Aida)

Despite knowledge of breast cancer and the mammography screening programme, it was apparent that major life stressors and competing priorities dominated their everyday lives. This aspect comprised both issues within the women’s everyday life in Denmark but also issues related to their ongoing engagement with social relations in their country of birth. All the women struggled daily with multiple diseases and health problems, many of them being chronic. Additionally, some women suffered from depression and one woman was living with the side effects after having surgery for a brain tumour. This naturally left little room for considerations of potential future diseases, such as cancer, therefore, early detection of breast cancer did not seem to be prioritised among the women. Simultaneously, competing priorities, such as maintaining relationships with relatives across the Danish borders, took up a great part of their everyday lives. The women were structurally embedded in transnational ties, through both emotional and financial obligations, which seemed to leave little room for participation in mammography screening.

### Transnational ties and the implication for participation in mammography screening

Maintaining relationships with relatives in country of birth and other countries where relatives had settled down seemed important for all of the women and many worked hard to maintain them. During a discussion on why she did not participate in mammography screening, a Somali woman explained that she currently had multiple other issues to take care of in her everyday life. Not only did she have a congenital heart defect and asthma but she also voiced great concerns for her relatives in Somalia suffering from the civil war and the drought in the country wherefore they daily called asking for moral and financial help. The culmination of these issues left little time for self-care:

“I forget myself sometimes (…) I blame myself, because I have my medicine, I have a good doctor; I have all the necessary things. Wealth? [material goods] I do not have much, but I have all the necessary things, which will prevent me from many problems [like breast cancer], but I don’t use them. I don’t benefit from them”. (Sumaiya)

Later in the interview Sumaiya explained how the absence of her sisters who lived in England further distressed her:

“I miss my sisters, we were so close, and they never come here to visit me”. (Sumaiya)

In the midst of an everyday life focused on helping relatives in Somalia and maintaining contact to her sisters, Sumaiya did not have the surplus energy to prioritise early detection of breast cancer, although she found screening relevant and remembered having received the invitation.

Technology such as phone and Skype was one way to maintain ties with relatives. A Turkish woman explained how she spent most of her days attempting to maintain contact with her children both in Denmark and in Turkey:

“If my children do not visit me then I am all alone in my room, then I am not that well of course (…) I talk on the phone with my daughters in Turkey and even with my daughters here for almost 24 hours [a day]”. (Sila)

The interview took place in Sila’s run-down apartment where she was living alone. She could not speak Danish, voiced that she was unable to get out of her apartment, and felt lonely since her only Turkish friend in Denmark now had passed away. In the beginning of the interview Sila mentioned her children’s wellbeing as the most important element in her life and explained how she often lied to them when they called asking about her wellbeing in order not to make them worry. She would always explain that she was feeling well even though that was not the case. Later in the interview, Sila voiced how she spent her days worrying about her children and feeling desperately lonely not being able to visit them in Turkey:

“I cannot travel to Turkey, neither in Turkey [nor in Denmark] I can get around. I also have epilepsy so I cannot travel alone and I also do not dare being in a bus”. (Sila)

Many of Sila’s relatives in Turkey suffered from cancer and throughout the interview Sila explained her fear for the disease. Sila had attained screening once, but when we asked about why she did not participate in the screening programme regularly, she explained how she was more preoccupied with concerns of how to maintain contact with her children rather than taking care of herself:

“To be healthy… if I should tell you the truth, then I am sitting in the chair for 24 hours and turn on the television and then I cannot find any other solutions than to start to cry”. (Sila)

Similarly, in another interview, an Indian woman expressed how she several times had attempted to participate in the screening programme but always without success. She described her daily worries for her sick mother who was living alone in India:

“My mom she is almost 80, 80 plus she is, but she lives all alone, all alone (…) I call her every day: “Mom, are you alright? Did you take your medicines?” (…) Every day I can only think about what about in the evenings, if she is still alive, if she needs some help”. (Meenu)

She prioritised travelling to India to visit her aging mother even though she had made an appointment to get a screening mammogram:

“But then, last time when she [the doctor] called, I said, after being a bit more energetic, “okay give me a time”. So she says: “In December [you will get an appointment], special for you”, but I had travelled to India”. (Meenu)

Among all women it seemed that participation in mammography screening was counterbalanced with a culmination of current issues, which they had to deal with in their everyday life wherefore they often did not get to participate in the screening programme even though they found it relevant.

### Economic and legal restrictions

Economic and legal restrictions often limited the women’s ability to travel, as well as precluded the possibilities of reuniting with relatives in Denmark. In a group interview, two Turkish women reported that multiple family members living in Turkey either had died from cancer or were currently sick. They worried about their sick relatives. One of the women had not been to Turkey for three years due to economic difficulties. Certain laws further complicated the women’s possibilities to visit their relatives in other countries. For example, the lack of a Danish passport made it difficult for the Somali women to apply for a visa to enter other countries. In a group interview four women discussed their notions of increased incidences of breast cancer in Denmark and therefore the importance of participating in mammography screening. However, later in the interview, they articulated that their main concern was not the risk of getting breast cancer but that they were unable to travel to Somalia. They had been in Denmark between 11–16 years and since they were unable to return to Somalia, they voiced how they desperately attempted to maintain contact to relatives in the country. From phone calls they were able to get in contact to few relatives who could explain the current situation in the country and report whether everyone was alive. For them Somalia was still an essential part in their life and the fear for their relatives suffering from the civil war preoccupied much of their everyday life leaving little room for engaging in recommended health behaviours:

“We have also told you, many of our diseases from the beginning are caused by the fact that we miss our homeland. We cannot afford to travel and we do not have a proper passport we can travel with (….)”. (Waris)

Similarly, in an individual interview, a Somali woman whose children and grandchildren lived in Somalia and England expressed her great desire to travel to England to visit her daughter and granddaughter, who had been diagnosed with cancer. The situation was clearly affecting her greatly. Her lonely everyday life in Denmark was occupied with taking care of her paralysed ex-husband. Since her granddaughter was life threatening sick in England it became even more important for her to travel. In the presence of these dominant issues participating in mammography screening became of minor relevance.

The women unable to travel back to their country of birth further spent great resources on creating social relations with other people in Denmark in order not to feel isolated. Afareen, an Iranian political refugee, was only rarely able to meet few relatives in Dubai. Her father and brother were killed during the Iranian revolution and Afareen explained how important it was for her to regularly go to the drop-in centre for pensioners in Denmark in an attempt to create a social network:

“If I just sit at home and look at the walls I will go crazy. One has to do something. So, they [Pakistani and Indian women from the interview] might have children and husbands and they get a bit more active, we have nothing. I have to do something”. (Afareen)

She had an opportunistic mammography screening a few years ago, and was aware of the risks of breast cancer and the current screening programme. However, Afareen fought with current life stressors; arriving to Denmark she had suffered from extreme loneliness and had for many years fought to create social relations in Denmark wherefore she currently focused her everyday life on maintaining these relations in order to have an acceptable quality of life.

### Transnational ties and variations over time

Some women maintained their transnational ties by sending money to their relatives in order to help them in their daily lives. These financial obligations were most evident among those women who fled to Denmark from countries consistently affected by war or poverty such as Somalia, Iran and Palestine. For example, the women who fled from Somalia were greatly affected by the drought in 2011 when the interviews were carried out. They spent much time following the situation and worried about how they, with limited financial resources, could help their relatives as well as their extended family left behind. In the end of the interview Sumaiya for example explained the worsened situation in Somalia, emphasising how participation in mammography screening became a minor issue in comparison to helping her relatives. She expressed great concerns about their desperate situation:

“You feel bad when you can’t [send money] (…) I saw in the news, I was just listening to the news, then they told about a new phenomenon, or whatever it’s called, that the Somalis are selling their organs to the rich from America and Saudi Arabia, can you believe that? (…) I didn’t know, I cried, I couldn’t help it. They are so desperate”. (Sumaiya)

Overall, for the refugee women participating in mammography screening was not considered as important as their multiple current issues. They were focused on establishing a social life in Denmark while helping relatives in their country of birth who suffered from ongoing war and poverty. In contrast, some of the women coming to Denmark through family reunification discussed how their financial situation had changed. In a group interview with Indian and Pakistani women, only one woman still financially supported her relatives in India, others had stopped supporting family in South Asia:

“Our husbands, they came due to the reason that they wanted to earn money and then sacrifice the family, right? But now our parents-in-law they are deceased and their family has also become self-sufficient (…) so now we don’t do it anymore [send money] (…) but it was a hard time you can say (…) you economised on everything in order to send money back”. (Haleema)

Haleema was one out of two women who managed to participate in screening regularly. Most other women in her situation did not participate; confirming the complexity of influences on health behaviour since multiple other obligations and competing priorities continued to exist for these women, making it hard to prioritise screening.

## Discussion

To the authors’ knowledge, this is the first study that investigates transnational ties and their implications for participation in mammography screening in a European setting. The study illustrates the need to take into account migrants’ multi-layered and multi-sited social context in studies on their health behaviour. Major life stressors for the women within their daily lives, such as the ability to maintain transnational ties, seemed to leave little room for concerns about breast cancer and therefore seemed to contribute to their lower participation in screening.

The present study recognises that low participation rates in mammography screening among migrants may be related to language barriers, lack of support to translation of the invitation, transport to “screening site” and emotional support when waiting for the results [2,3]. Simultaneously, this study demonstrates that participation in screening cannot merely be explained by direct influences on behaviour occurring in the country of destination [7]. Other studies have attempted to explain the low participation rate from religious and fatalistic beliefs [24,25]. As the present study illustrated, the women had a positive attitude towards the Danish healthcare system and were in regular contact with their general practitioner. Early detection of breast cancer was easier to de-prioritise in comparison to current diseases and other immediate stressors such as care for sick relatives and maintaining social relations. However, it is important to stress that the women *did* care about themselves health wise and coped with different kinds of diseases.

The study shows that to further understand migrants’ screening behaviour we need to take into account their spatially extended social context. Migrants are engaged in a dynamic social life beyond one geographic location [17,26] and their transnational ties seem to be of great importance in understanding their health behaviour and participation in mammography screening. Maintaining contact with relatives living in other countries seemed to occupy the women and they spent much time talking to relatives on the phone or through Skype since they often were not able to visit them. Some of the women further maintained their ties through financial obligations and these women seemed to work hard every day in order to take care of their suffering relatives. Their own health issues were counterbalanced with such life stressors, and consequently, participation in mammography screening, although perceived as relevant, seemed to be de-prioritised.

Most migration scholars recognise the impact of transnational ties in migrants’ everyday lives [17,26,27], but there is a consistent need for it to gain more attention within health behaviour literature. No European studies and only a few American studies have addressed this aspect in relation to migrants’ participation in mammography screening [9,16]. Not only should research broaden its perspectives from only focusing on psychological factors, lack of knowledge or religious and fatalistic beliefs. Future research should also recognise that many individuals’ social lives are not confined by nation-state boundaries [18]. Migrants’ integration into a receiving country and transnational ties to relatives in their country of birth can occur at the same time and reinforce one another [18]. Therefore, migrants’ life trajectories and multi-layered, multi-sited social ties should be embraced in studies on migrants’ health behaviour.

Researchers may discover that migrants’ health behaviour may also be explained partially by the fact that their own health behaviour is counterbalanced with other obligations and major life stressors. It is important to recognise that the influence of context is by no means limited to migrant populations but rather is an important factor among all socially marginalised groups. However, for migrants, the social context differs from that of other groups since it is more likely to transcend national borders.

Importantly, migrants’ transnational ties are dynamic and change over time. The present study illustrates how sudden changes within the women’s country of birth influenced their everyday lives in Denmark and thus may influence their motivation for screening. At the time of the interviews the Somali women were greatly affected by the drought in Somalia causing widespread hunger. They closely followed the situation and helped their relatives as much as they could. In contrast, the women who arrived many years ago from countries such as Turkey or Pakistan due to family reunifications increasingly experienced that the relatives they left behind had become self-sufficient. In some cases, these women now received financial support from their relatives. Future studies should further investigate the impact of transnational ties on migrants’ health behaviour with emphasis on differences among subgroups among migrants as well as dynamics over time.

This study by no means covers every aspect of transnational ties that may be of significance to migrants’ health behaviour but it does contribute to the development of new frameworks of study from which the contextualisation of migrant’s health behaviour may be examined in greater depth and with the needed methodological rigour such as multi-sited studies.

The study indicates that to increase participation in mammography screening among migrant women it is inadequate merely to translate the invitation letter to screening into migrant women’s native languages in an attempt to bridge language barriers while not take into account various stressors influencing women’s everyday life. In accordance with the present study it is further important to sufficiently understand women’s ability to and motivation for participating in mammography screening programmes in a context that reaches beyond national borders. To attain ethnic equality in public health programmes it seems necessary to integrate social and public health efforts and work community-based to empower migrant women to speak for and act upon their needs.

### Methodological reflections

It is important to recognise the diversity within the study population as well as the relative small number of interviewees. The present study is explorative and calls for additional research within the field of migrant’s transnational ties and the influence on their health behaviour. Reasons for and experiences with migration as well as time residing in Denmark differed among all of the women naturally resulting in diverse life stories. The women who fled to Denmark recently as refugees seemed strongly connected to their country of birth and struggled with maintaining ties to relatives who were often threatened to die. Women who arrived to Denmark long time ago from countries currently unaffected by war seemed more settled in Denmark but still struggled with maintaining emotional ties to their relatives. Women also differed regarding household status naturally influencing their social relations in Denmark. Despite the diversity of the study population there were certain shared common characteristics among the women. All were migrants living in socially disadvantaged neighbourhoods and are categorised as socially marginalised in accordance with the objective criteria of the study. In Danish society this group of people have suffered from rather negative media coverage of migrants in recent years. To some extent it might be difficult to disentangle these factors associated with being poor and marginalised from being "transnational". Despite the categorisation as socially marginalised most women were advantaged in various ways; they spent a great deal of resources taking care of relatives located both in Denmark and in other countries and managing their own multiple diseases.

The use of interpreters during interviews challenges the interaction between interviewer and interviewees [28]. However, interpreters were informed of research ethics and guidelines for interpretation prior to interviews, and were overall contributing to a trustful and nuanced interaction. Validity of analysis was enhanced by analysing the data individually followed by comparisons of emerging themes, and by presenting intermediate findings to multidisciplinary research groups.

## Conclusion

The present study emphasises the need to contextualise health behaviour by acknowledging the influence of migrants’ transnational ties on their ability to and motivation for participating in mammography screening programmes. Public health interventions targeting migrants should therefore take into account the influence of transnational ties on migrants’ health behaviour.

## Competing interests

The authors declare that they have no competing interests.

## Authors’ contributions

LLK and MK designed the study and carried out the interviews and analysis. LLK drafted the manuscript in cooperation with MK. MN, ABK and AM participated in the design of the study and helped to draft the manuscript. The Psychosocial Research Committee, Danish Cancer Society, funded the study. The research team is independent of the funders and the views expressed are those of the researchers, not the funding body. The study adheres to the RATS guidelines on qualitative research. All authors read and approved the final manuscript.

## Pre-publication history

The pre-publication history for this paper can be accessed here:

http://www.biomedcentral.com/1471-2458/13/431/prepub
